# Assessment of Essential Newborn Care Services in Secondary-level Facilities from Two Districts of India

**Published:** 2014-03

**Authors:** Sumit Malhotra, Sanjay P. Zodpey, Aishwarya L. Vidyasagaran, Kavya Sharma, Sunil S. Raj, Sutapa B. Neogi, Garima Pathak, Abhay Saraf

**Affiliations:** ^1^All India Institute of Medical Sciences, New Delhi, India; ^2^Indian Institute of Public Health-Delhi, Public Health Foundation of India, New Delhi, India

**Keywords:** Clinical competence, Health facilities, Health personnel, Newborn care, Process assessment, India

## Abstract

India faces a formidable burden of neonatal deaths, and quality newborn care is essential for reducing the high neonatal mortality rate. We examined newborn care services, with a focus on essential newborn care (ENC) in two districts, one each from two states in India. Nagaur district in Rajasthan and Chhatarpur district in Madhya Pradesh were included. Six secondary-level facilities from the districts─two district hospitals (DHs) and four community health centres (CHCs) were evaluated, where maximum institutional births within districts were taking place. The assessment included record review, facility observation, and competency assessment of service providers, using structured checklists and sets of questionnaire. The domains assessed for competency were: resuscitation, provision of warmth, breastfeeding, kangaroo mother care, and infection prevention. Our assessments showed that no inpatient care was being rendered at the CHCs while, at DHs, neonates with sepsis, asphyxia, and prematurity/low birthweight were managed. Newborn care corners existed within or adjacent to the labour room in all the facilities and were largely unutilized spaces in most of the facilities. Resuscitation bags and masks were available in four out of six facilities, with a predominant lack of masks of both sizes. Two CHCs in Chhatarpur did not have suction device. The average knowledge score amongst service providers in resuscitation was 76% and, in the remaining ENC domains, was 78%. The corresponding average skill scores were 24% and 34%, highlighting a huge contrast in knowledge and skill scores. This disparity was observed for all levels of providers assessed. While knowledge domain scores were largely satisfactory (>75%) for the majority of providers in domains of kangaroo mother care and breastfeeding, the scores were only moderately satisfactory (50-75%) for all other knowledge domains. The skill scores for all domains were predominantly non-satisfactory (<50%). The findings underpin the need for improving the existing ENC services by making newborn care corners functional and enhancing skills of service providers to reduce neonatal mortality rate in India.

## INTRODUCTION

Worldwide, out of the total 133 million livebirths, 3.7 million die in neonatal period. Another 3 million are stillborn.  Ninety-eight percent of deaths take place in the developing world, where 90% of babies are born. Of these deaths, 76% or 3 million occur in early neonatal period. The risk of deaths in the neonatal period in developing countries is over 7 times greater than in developed countries (1). Also, most of the neonatal deaths occur in Asian countries, more notably in India, which accounts for more than a quarter of the global neonatal mortality (2). The current neonatal mortality rate (NMR) in India is 31 per 1,000 livebirths (3). Infections, birth asphyxia, and prematurity/low birthweight (LBW) are major causes of deaths within this period (4). Interventions combining resuscitation of newborn baby, breastfeeding, prevention and management of hypothermia and kangaroo mother care (KMC) can reduce NMR by more than half (5). It is vital that health systems are equipped with necessary supplies, at least heating source, resuscitation bags and mask, and mucus devices. Equally important is that the staff members are competent enough to provide essential newborn care to maximize gains from proven evidence-based and cost-effective interventions to combat the burden of neonatal morbidity and mortality in India.

A lot of thrust has been imparted in recent years by the Government of India to improve facility-based newborn care interventions for reducing NMR in the country. We carried out an assessment of newborn care services, with focus on essential newborn care (ENC) in two districts of two Indian states with high neonatal disease burden in 2010. The questions that this study sought to answer were : (i) what kind of ENC services were provided at secondary-level study facilities?; (ii) whether facilities had necessary infrastructure, human resources, and other supplies for rendering ENC services (whether all equipment needed for providing ENC services were available and functional)?; and (iii) whether service providers involved in rendering newborn care possessed adequate knowledge and skills in different domains of ENC? At the time of assessment, no special programme was in place to improve facility-level newborn care in these two districts. The overall purpose of the assessment was to ascertain the current situation of ENC so as to find out the strengths and weaknesses in the quality of newborn care service provision, and the findings would inform programme managers to strengthen the newborn care delivery in their service area. The ENC components included in the assessment were resuscitation, warmth, breastfeeding, and prevention of infections (6).

## MATERIALS AND METHODS

The study was conducted in two states: Madhya Pradesh [NMR 44/1,000 livebirths (7)] and Rajasthan [NMR 40/1,000 livebirths (7)]. From each state, one district was chosen: Chhatarpur [NMR 51/1,000 livebirths (7)] in Madhya Pradesh and Nagaur [NMR 42/1,000 livebirths (7)] in Rajasthan. The districts selected were primarily naïve in initiating any form of facility-based newborn care interventions and had high institutional delivery load. The selection of districts was guided through central and local government officials. From each of the selected districts, three facilities—one district hospital (DH) and two community health centres (CHCs)—were sampled and these facilities combined were catering to maximum institutional deliveries within the district. The study facilities included were rendering secondary level of care. The CHCs usually cater to a population of 80,000 to 120,000, have 30-50 inpatient beds, and maternal child services are provided by specialist doctors in obstetrics and paediatrics. The district hospital usually caters to more than 0.5 million people in India and has maximum concentration of specialist doctors in the government system. The DH serves as a major referral centre within the district-based healthcare system in India. Apart from medical doctors, the secondary-care facilities have nursing staff in the form of staff nurses with three to four years of professional education entrusted with work relating to labour room and ward duties within the hospital. The other cadre comprises auxiliary nurse-midwives (ANMs) with one to two years of professional education, who, apart from performing hospital duties in reproductive and child healthcare, are also entrusted with community-based outreach activities.

The ENC situation assessment had several components: record review for ascertaining delivery load and neonatal service provision, facility observation, and competency assessment of providers in essential newborn care. The tools used were developed and finalized for the study following a consultative meeting with experts in neonatal care.

Data on the services delivered at the facilities in relation to newborn care, such as number of births, admissions, and referrals, were collected from registers maintained at the facilities. Details of birth, including number of livebirths and stillbirths were recorded from labour room registers. Other details regarding neonatal admissions and deaths were collected from registers maintained in the paediatric wards. These data were collected month-wise for a period of one year from April 2009 to March 2010.

The study facilities were assessed for provision of ENC services in terms of availability of infrastructure, human resources, support services, and equipment. The labour rooms, operation theatres (OTs), and paediatric wards were examined for the presence of Newborn Care Corner (NCC). This was defined as a space within the labour room or OT for providing immediate newborn care to all newborns. Ideally, NCC should be within the labour room and be equipped essentially with radiant warmer as a source of heating and resuscitation-kit for reviving asphyxiated neonates (8). A structured checklist was used for assessing the facility situation for the provision of ENC.

The knowledge and skills assessments were carried out for personnel rendering newborn care—medical doctors, including specialists (paediatricians and gynaecologists) and nursing staff [staff nurses and auxiliary nurse midwives (ANMs)]. These care providers ranged from contractual to regular staff members in these facilities, with varying years of service, some recently employed to some with more than five years of service. None of the providers was included in any special newborn care training programme as part of in-service training at the time of assessment. Doctors working as paediatricians and gynaecologists were assumed to receive newborn care training as part of their professional basic education. Various providers were included in the assessment so as to ensure representativeness of the sample and to assess real working scenario in secondary healthcare facilities in the study districts. The knowledge and skills assessment tool used for this study were endorsed by Indian Academy of Paediatrics. The knowledge assessment methods included a self-administered written questionnaire in either English or Hindi language. Forty true or false questions were arranged in two sets to cover five domains, viz. neonatal resuscitation, infection prevention, care at birth, hypothermia prevention, KMC, and breastfeeding. The first set comprised twenty questions, exclusively testing knowledge on neonatal resuscitation. Skills assessments were performed through direct observation of demonstrations carried out on a baby mannequin with all required equipment made available. Summary of the key skills assessed are presented in the appendix. These were objectively assessed using performance evaluation checklists, a technique that has been found reliable and valid in testing skills relating to newborn resuscitation (9). Two performance checklist sets were used, the first focusing on newborn resuscitation and the second on other ENC domains. Skills were assessed by lead investigator of the study (SM) at both the study districts, who was trained as master trainer in ENC by the Government of India.

Descriptive analysis was carried out and expressed with proportions and averages, using Microsoft Excel Office 2007 and STATA (version 10.0). Knowledge and skills assessed were graded as satisfactory (more than 75%), moderately satisfactory (50-75%), and not satisfactory (less than 50%), based on the percentage of correct answers in each domain.

**Table 1.** Live- and stillbirths in the study facilities (April 2009–March 2010)

Study facility	Livebirth	Stillbirth	Stillbirth rate (per 1,000 births)
DH, Nagaur	4,548	176	37.3
DH, Chhatarpur	6,514	257	38.0
CHC, Kuchaman city	1,967	63	31
CHC, Merta city	1,234	19	15.2
CHC, Badamalhera	1,988	43	21.2
CHC, Nowgong	3,162	89	28.2

CHC= Community Health Centre;

DH=District Hospital

### Ethics

Written informed consent was taken from all healthcare providers, and performance was assessed maintaining confidentiality. The ethical approval was obtained from Institutional Ethics Committee of Public Health Foundation of India.

## RESULTS

### Facility-level births and provision of neonatal care

There were 4,548 (monthly range 248 to 515) and 6,514 (monthly range 377 to 748) livebirths recorded between April 2009 and March 2010 at the district hospitals in Nagaur and Chhatarpur respectively. The recorded stillbirths included both fresh and macerated stillbirths, and the stillbirth rate of both the DHs was around 38/1,000 births. The median annual births in the study CHCs was 1,978, with a range of 1,234-3,162 and more births taking place at Chattarpur CHCs. The stillbirth rate in these facilities ranged from 15 to 31/1,000 births (Table 1).

**Figure 1.**
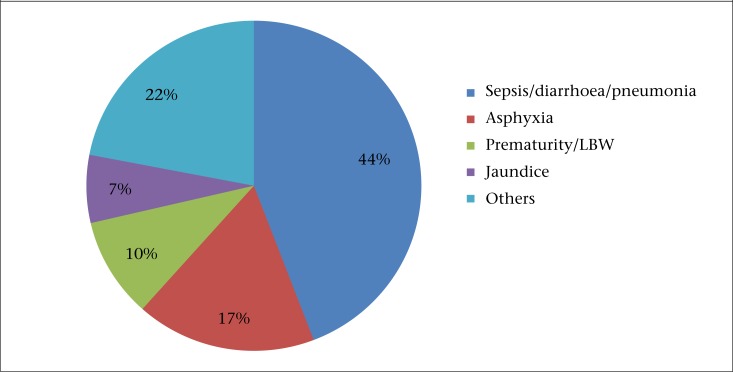
Causes of neonatal admissions at district hospitals (April 2009–March 2010)

**Figure 2.**
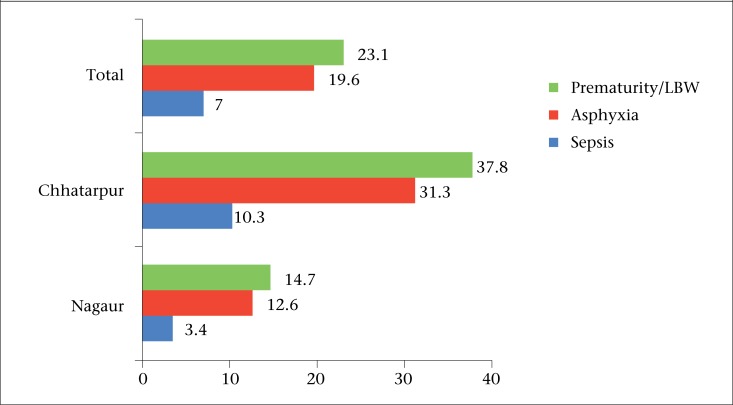
Cause-specific case-fatality rate per 100 admissions in the district hospitals under study

Neonates requiring special care were admitted in the paediatric ward in district hospitals only. No neonates were admitted at the study CHCs at the time of the study. While neonates requiring specialized care were referred to other facilities, these referrals were not recorded at the CHCs and, so, these data were not available for review at the time of this study. There were 1,114 and 992 neonatal admissions in Nagaur and Chhatarpur district hospital respectively. Majority (74%) of the admitted neonates were aged less than one week (Table 2). The registers did not note if the admitted neonates were born within the facility or referred from outside. The causes of neonatal admissions and deaths are shown in Figure 1 and Table 2. The most common neonatal morbidity was sepsis in both the districts (40% in Nagaur and 50% in Chhatarpur), followed by birth asphyxia. There were a total of 75 neonatal deaths in Nagaur District Hospital and 144 deaths in Chattarpur District Hospital during the study period. The most common cause of deaths was birth asphyxia (39%) in Nagaur and sepsis (35%) in Chhatarpur. The cause-wise case-fatality rate per 100 admissions is presented in Figure 2. Maximum case fatality was observed for premature babies, followed by those who were admitted with birth asphyxia.

**Table 2.** Details of neonatal admissions and deaths at the district hospitals under study (April 2009–March 2010)

Parameter	District Hospital, Nagaur	District Hospital, Chhatarpur
Neonatal admissions	1,114	992
Timing of admissions: n (%)		
Within 24 hours	476 (43)	137 (14)
1-7 day(s)	340 (30)	609 (61)
8-28 days	298 (27)	246 (25)
Causes of admissions: n (%)		
Sepsis/diarrhoea/pneumonia	440 (39.5)	493 (49.7)
Birth asphyxia	229 (20.6)	137 (13.8)
Prematurity/LBW	129 (11.5)	74 (7.5)
Jaundice	74 (6.6)	66 (6.7)
Others	242 (21.7)	222 (22.3)
Neonatal deaths	75	144
Causes of deaths		
Birth asphyxia	29 (38.7)	43 (30)
Prematurity/LBW	19 (25.3)	28 (19.4)
Sepsis/diarrhoea/pneumonia	15 (20)	51 (35.4)
Others	12 (16)	22 (15.3)

### Readiness of facilities for the provision of ENC services

#### Infrastructural facilities and support services

At the time of this assessment, both the DHs at Nagaur and Chhatarpur did not have separate functional units for newborns but, within the paediatric ward, each of the two beds with radiant warmers was used for providing newborn care. Sick neonates were admitted and cared for within the paediatric wards. Newborn care corners were present in the facilities. These were situated in spaces adjacent to the labour room, except in two CHCs, one from each study district where it was situated inside the labour room. In the surveyed facilities where operation theatres were functional with ongoing caesarean sections, only trolleys or trays were used for handling newborns soon after delivery.

Dedicated and demarcated spaces within the labour rooms were seen more in facilities in Chhatarpur than those in Nagaur. In two out of three facilities in Nagaur, NCCs were not used and maintained poorly. Instead, the common practice was to use the tray in the labour room for holding the baby and keeping on a hard surface for performing resuscitation techniques. Only in half of all facilities, the NCC was kept draught-free. Wall clocks were fitted to record the time of birth in all facilities, except one of the CHCs in Chhatarpur. Medical thermometers were available in about half of the facilities but none of the facilities was equipped with room thermometers.

Other basic physical facilities relating to newborn care, such as cord-tie, cord-cutter, and infant-weighing scale were available at most facilities, except in one of the CHCs in Nagaur. Supply of clean towels was meagre, and the practice of wrapping the neonates in clothes brought by the mother or other attendants was common. A similar situation was observed with regard to hand-gloves. Other items for asepsis, such as disinfectants, disposable syringes and needles, gowns, and slippers were available and being used in most facilities.

#### Equipment and support services for essential newborn care

The hospitals were assessed for availability and functioning of equipment needed to provide essential newborn care as well as equipment to treat sick infants, like feeding-tubes, IV infusion sets, and phototherapy units. Equipment for newborn resuscitation were available and functioning in most of the study facilities. Resuscitation bags with masks were available in four out of six facilities. However, masks of different sizes were not available. In one of the CHCs at Chhatarpur, mouth-to-mouth breathing was used for newborn resuscitation as an alternative to bag and mask ventilation as and when required.

Oxygen supply was present in most facilities, and suction devices were present and functional in all facilities, except the two CHCs in Chhatarpur. In these facilities, a locally-prepared suction device, made by cutting intravenous (IV) tube, was in use. Laryngoscopes and endotracheal tubes for infants were available at the DH in Nagaur but not in Chhatarpur. At the CHC level, even if available, these were not being used.

Radiant warmers for infants were being used in the labour rooms as well as the paediatric wards. Within the labour room, these warmers were available in only three facilities (all in Chhatarpur) but were functional only in two. The warmers were also available and functional in both district hospitals in their paediatric wards. Regular inspection and maintenance of these equipment were not carried out, and delay in reporting of repair workers was a frequent problem.

Cups and spoons for feeding the newborn were not available in any of the facilities, and nasogastric tubes for feeding sick infants were available only at the district hospitals. About half of the facilities had paediatric infusion sets, available either in the labour room or operation theatre. Almost all assessed facilities did not have a phototherapy unit. The one available in the Chhatarpur District Hospital was not in working condition.

#### Essential drugs and supplies

The facilities were assessed for the availability of basic antibiotics, like ampicillin and gentamicin; emergency drugs, like adrenaline and aminophylline, IV fluids; and other essential drugs, like phenobarbitone and vitamin K. Most drugs and IV fluids were available in all facilities at the time of assessment. Only anticonvulsants, like phenobarbitone were not available. Registers maintained at the facility revealed sufficiency of stock with few stock-outs in the past one year.

#### Human resources

All facilities were assessed for the availability of trained personnel in the labour room or operation theatre. It was noted that all facilities had medical officers, staff nurses, and ANMs to provide newborn care services, with paediatricians usually being available on call. Medical officers and staff nurses/ANMs were available in all facilities to provide round-the-clock services. However, most deliveries were handled by staff nurses/ANMs, and they were largely responsible for rendering immediate essential newborn care services. All facilities had at least one trained obstetrician to attend deliveries.

Among all the health facilities surveyed, paediatricians were posted in five out of six facilities, except one CHC. In district hospitals, paediatricians were called to provide care in an event when the attending staff nurse/ANM was not able to resuscitate a newborn. In facilities where paediatricians were not present, especially at CHCs, immediate referral to other facilities was done.

### Competency assessment of healthcare providers in ENC

A total of 38 healthcare providers, 19 each from Nagaur and Chhatarpur district, were assessed. Among them, 14 were doctors (9 specialists and 5 general-duty medical officers), and 24 providers belonged to nursing staff categories (15 staff nurses, 9 ANMs). The individual domain and category-wise average scores are presented in Table 3. It reveals that, in most of the domains, knowledge and skill scores were found to be higher or similar in doctors when compared with nursing staff, except for skill domains relating to preparation at birth and breastfeeding, where nursing staff scored higher than doctors. Table 4 presents the results in different domains based on grading of knowledge and skills into three categories. Majority of the providers scored moderately satisfactory scores for most of the knowledge domains, except KMC and breastfeeding where it was largely satisfactory. The skill scores for all domains were predominantly non-satisfactory.

**Table 3.** Average scores (%) in different domains of essential newborn care

Category of health personnel	Resuscitation		Infection prevention and care at birth		Hypothermia prevention		Kangaroo mother care		Breastfeeding	
	K	S	K	S	K	S	K	S	K	S
Doctors	80	32	73	25	77	33	83	25	94	36
Staff nurses	74	24	68	41	63	33	82	26	87	53
ANMs	75	13	81	31	72	24	82	11	98	63

K=Knowledge,

S=Skill,

ANMs=Auxiliary nurse-midwives

**Table 4.** Knowledge and skill grade of healthcare providers (n=38) in ENC domains

Domain		Satisfactory n (%)	Moderate n (%)	Non-satisfactory n (%)
Resuscitation	K	17 (45)	21 (55)	0
	S	0	3 (8)	35 (92)
Infection prevention and care at birth	K	14 (37)	24 (63)	0
	S	0	13 (34)	25 (66)
Hypothermia prevention	K	17 (45)	19 (50)	2 (5)
	S	0	2 (5)	36 (95)
Kangaroo mother care	K	23 (61)	13 (34)	2 (5)
	S	2 (5)	10 (26)	26 (68)
Breastfeeding	K	30 (79)	8 (21)	0
	S	5 (13)	18 (47)	15 (39)

K=Knowledge,

S=Skill,

satisfactory (domain score more than 75%),

moderately satisfactory (50-75%),

and not satisfactory (less than 50%),

based on the percentage of correct answers/performances in each domain

**Figure 3.**
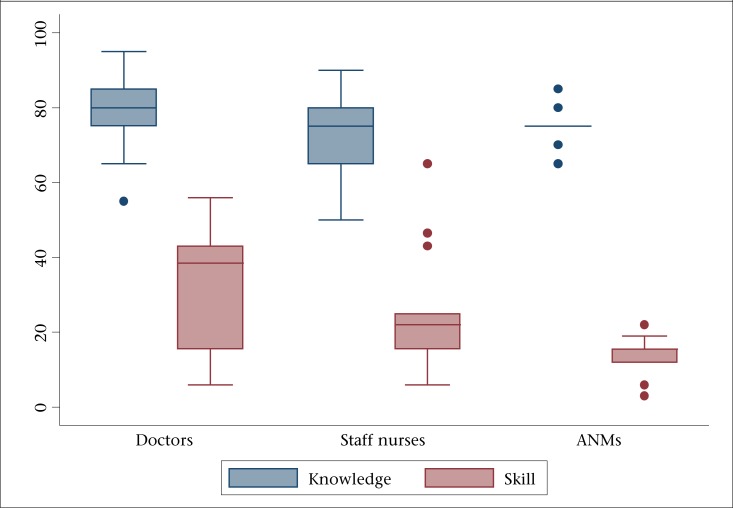
Median and interquartile range (%) for neonatal resuscitation-related knowledge and skill according to category of personnel assessed

Overall, the mean average score for knowledge and skills in neonatal resuscitation was 76% (SD±10) and 24% (SD±15). The mean score for all doctors on resuscitation-related knowledge and skill was 80% (SD±11) and 32% (SD±16) respectively. For the nursing staff, the corresponding average score for knowledge and skill was found to be 74% (SD±10) and 20% (SD±14). The medians and interquartile range for the same are presented in Figure 3, reflecting contrast in high knowledge and poor skill relating to neonatal resuscitation. Doctors performed better than other categories of care providers. Among the doctors, all specialists (n=9) had satisfactory knowledge than general-duty medical officers (n=1). In Nagaur district, 80% (4/5) doctors had satisfactory knowledge compared to 77% (7/9) in Chhatarpur. Similarly, higher proportion of nursing staff had satisfactory knowledge (93%, 13/14) in Nagaur than Chhatarpur (40%, 4/10). In skills assessment among all types of care providers, 77% (7/9) doctors in Nagaur and 90% (9/10) nursing staff in Chhatarpur demonstrated unsatisfactory performance; 79% (n=29) of the providers performed suction in the event of baby not crying, 61% (n=23) did both mouth and nose clearing, and only 8% (n=3) did this in correct order. There were critical lapses in terms of checking function of the bag and mask, using it, and taking corrective actions if chest-rise was not adequate after initial application of the bag and mask.

Domains in essential newborn care other than resuscitation were tested using a second set of knowledge test and performance evaluation checklist. In this set, the overall average knowledge and skill score was 78% (SD±10) and 34% (SD±12). The average knowledge score for doctors was 81% (SD±11) and, for nursing staff, it was 77% (SD±9). However, the average skill score for doctors were 31% (SD±13) and, for nursing staff, it was 35% (SD±12). Only 8% (n=3) of the providers knew correctly the six steps of handwashing and its duration. Although 89% (n=34) of the providers demonstrated wiping of newborn with dry cloth, 63% (n=24) showed putting neonate on warm surface, and only 5% (n=2) were able to use all methods to prevent heat loss. Nearly two-thirds of the care providers demonstrated partial correct method to measure the temperature; correct positioning of thermometer and timing were largely unknown; 63% (n=24) of personnel were not able to place mannequin for practising KMC; 76% (n=29) of the care providers demonstrated all four steps correctly in positioning the baby for breastfeeding.

In these domains, no major differences were found among personnel working in two study districts, except that the average knowledge score for “infection prevention and care at birth” was higher in Chhatarpur (63% vs 53%), KMC was higher in Nagaur (68% vs 53%), and skill scores for KMC were lesser in Nagaur (89% vs 47%). For all the domains tested, knowledge scores were similar when compared between district hospitals (DH) and CHCs functionaries whereas, for skills, personnel of the district hospitals performed better than those posted at CHCs (33% vs 18% for resuscitation and 40% vs 29% for other domains).

## DISCUSSION

This study is a cross-sectional assessment that examined the essential newborn care services in two districts with high neonatal and infant mortality rate. The study districts had usual number of institutional deliveries as being reported by many other districts of the country (10). The stillbirth rates at the level of district hospitals and CHCs were on the higher range. India tops among the high-burden countries contributing to maximum stillbirths globally. It was estimated that, every year, 613,500 stillbirths at the third trimester occurred with a rate of 22 per 1,000 births (range 17-36). Also, inter-state variation prevails within the country with high rates from central India, reaching up to 66 per 1,000 births (11). Amongst the health facilities studied, inpatient neonatal care was rendered by DHs only. At CHCs, no admissions were done, despite presence of paediatricians; this policy of no neonatal admissions at the level of CHCs has been noted in earlier assessments also (10), and this is an area requiring concerted efforts to manage common neonatal morbidities at this level. Sepsis, birth asphyxia, and prematurity/LBW were the most common neonatal morbidities handled; similar results were reported earlier also (12).

The study facilities had newborn care corners either within the labour room or adjoining room, although this was not utilized in all. Most of the essential equipment were found at the district level but, at the CHCs, there were deficiencies noted in regard to availability. One of the CHCs in Chhatarpur did not have bag and mask for resuscitation that resorted to mouth-to-mouth breathing for reviving asphyxiated neonates. Masks of both the sizes were absent in four out of six facilities. Suction device was also not present in two CHCs of Chhatarpur. Radiant warmer was either not there in the facilities or was not in functioning state. Rapid assessment of essential newborn care services in 11 districts from 10 states conducted in 2009 reported low availability of resuscitation area in sampled facilities (64% in DHs and 46% in CHCs). This also reported that most of the ENC equipment were available in all DHs but only in two-thirds of CHCs (10). A programme evaluation of *Janani Suraksha Yojana*, a conditional cash transfer scheme to promote institutional deliveries in India that included both the study states reported suboptimal neonatal care in terms of availability, functionality, and usage of newborn care equipment (13). A recent study reported infrastructure in relation to essential newborn care services in 13 CHCs from Bharatpur district of Rajasthan and found deficiencies in the presence of equipment. Only 3 out of 13 (23.1%) had radiant warmers, 4/13 (30.8%) had resuscitators, and 9/13 (69.2%) had suction pumps available in the facilities. None of the included CHCs in this assessment had fully-equipped newborn care corner (14). Reports of supervisory visits to certain districts of Rajasthan by central government personnel also highlighted lapses in essential newborn care provision in Dholpur and Bharatpur districts (15,16). The recent reviews of the National Rural Health Mission in India in 2010 and 2011, a major primary-care reform initiative launched to bring about architectural correction in infrastructure and human resources in all public health facilities also pointed out lacunae that exist in the availability of functioning equipment and also in the use of newborn care corners (17,18). This assessment included Barmer and Chittaurgarh districts of Rajasthan state. In Barmer, most labour rooms did not have newborn care corner. In Chittaurgarh, the equipment for newborns were recently supplied to the facilities. Absence of simple lifesaving equipment for rendering ENC points out to serious gap in the provision of quality care. CHCs require special attention in this, and lacunae in the availability of equipment require serious rectification. It was also observed that, despite presence of newborn care corners, these were not optimally utilized. This calls for a change of mindset and provision of adequate sensitization of care providers using the newborn care corners.

Most of the normal deliveries were handled by nursing staff, especially at the level of CHCs. Although the study facilities had obstetricians and paediatricians, they were called only when needed. It was imperative that all these personnel involved in delivery care should possess adequate competencies to provide essential newborn care. The current study underscored the critical gap that existed in competencies of healthcare providers in neonatal resuscitation and essential newborn care. There were no major differences between doctors and other categories of health personnel in the second set comprising essential newborn care knowledge and skill components but, for neonatal resuscitation, doctors demonstrated higher competencies than other health personnel assessed. The median knowledge and skill score for doctors was respectively 5 and 23 points higher. Knowledge was the lowest for doctors in the domain of preparation and essential care at birth and for nursing staff in the domain of hypothermia prevention.

Although knowledge was found satisfactory in most of the domains, there existed a huge deficiency in performance skills among all cadres of personnel assessed. Resuscitation, care at birth, and KMC were the weakest areas. Personnel assessed had little or no experience in using these skills. In one of the facilities included, there was no bag and mask, and the personnel there used mouth-to-mouth breathing when required. In other two facilities, the bag and mask were present but were not in use. Also, there was over-dependency among nursing staff towards doctors for using skills relating to resuscitation, even though maximum deliveries in these facilities were conducted by nursing workforce. Surprisingly, in the domains of “breastfeeding, infection prevention, and care at birth”, nurses demonstrated better skill scores than doctors. This could be due to the fact that nurses were assuming larger responsibility in counselling mothers for breastfeeding and handling higher volumes of cases with normal labour in these facilities. Also, it was found that staff posted in district hospitals had better skills than rest of the health facilities, which can partially be attributed to higher exposure to load in terms of volume of births and larger opportunities for using skills on regular basis. Another reason for this may be more concentration of specialist doctors at the district hospitals compared to CHCs.

This is one of the few studies in Indian setting, assessing competencies of health personnel in providing ENC. A rapid assessment of ENC conducted in 11 districts across 10 states in the country reported that a sample of 44 doctors in the fields of neonatal resuscitation (41%), hypothermia prevention (61%), breastfeeding (34%), and infection prevention (23%) had both satisfactory knowledge and skills. The study pointed out limited capacity of staff nurses and ANMs in having sufficient knowledge and skills in neonatal resuscitation (41%) and hypothermia prevention (52%) (10). The study employed different methods in assessing knowledge and skills from what is used in the present study. A similar study conducted among medical officers, lady health visitors, and workers of Pakistan reported poor performance in resuscitation skill, and only 50% demonstrated correct steps of immediate newborn care (19). Low competencies in newborn care among service providers have also been reported consistently from other settings (20-22). Studies that have reported the effect of training in newborn resuscitation recorded initial low scores (average skill scores usually less than 50%) in essential care and resuscitation, that significantly improved after the capacity-building efforts (23-25). One of the key strengths of this assessment is the fact that it presents a rapid methodology for monitoring essential newborn care in a comprehensive way and will allow replication in other settings for improvising the newborn services in district health systems.

### Limitations

There are some limitations of this assessment. The sample tested is small, taken from six health facilities across two districts. This might not be generalizable to other districts but, seeing consistent results from similar assessments carried out, we believe that, currently, essential newborn care provision requires improvement in most of the settings in India. The functionality of newborn care corners with all available equipment is low in many settings with high disease burden as discussed previously, and competencies in resuscitation and essential newborn care are currently poor and will apply to providers of other facilities in local context. Also, the knowledge questionnaire in the current study employed true or false type of questions. There might be high positive results in this mode but the skill assessment in the form of performance evaluation checklist has been shown to be a reliable and valid tool of assessing neonatal resuscitation skills (9).

### Conclusions

The study highlighted an urgent need for having skill-based capacity-building initiatives targeted to health personnel posted in facilities where childbirth occurs and immediate care are provided to the neonates. Availability of functional bag and mask in these facilities will also be critical to practise the acquired skills in resuscitation. Availability and functionality of necessary equipment with optimal skills to use the equipment will be important to realize the potential gains that can be achieved through provision of ENC. Components of essential newborn care and neonatal resuscitation are proven interventions for reducing NMR and stillbirth rate; it is imperative that providers be equipped and made competent to practise these skills that can accelerate the progress towards achieving Millennium Development Goal 4 relating to child survival. After correcting deficiencies in the study districts and providing skill-based training, it is also recommended that such periodic assessments be undertaken for improving the existing situation further.

## ACKNOWLEDGEMENTS

The study was supported by Ministry of Health and Family Welfare, Government of India. We are thankful to the officials from State Government of Madhya Pradesh and Rajasthan, Chhatarpur and Nagaur district officials for cooperating and providing consent for undertaking this study. We are also thankful to the experts who reviewed our tools and helped us in their finalization. We are also indebted to the staff members of facilities, who have given their valuable time in the data-collection procedures.

**Appendix.** Summary of key skills for competency testing in different ENC domains

Resuscitation
✓ Demonstrating correct steps in right sequence for applying positive pressure ventilation
✓ Testing function of bag and mask
✓ Assessment of baby's breathing and repositioning, if required
✓ Indicating need for positive pressure ventilation
✓ Positioning the head and applying face mask correctly
✓ Taking corrective action if chest-rise is not adequate
✓ Ventilating for adequate time (30 seconds), with appropriate rate and pressure (40-60 breaths/minute)
✓ Action based on heart rate if not breathing well
**Infection prevention and care at birth**
• Demonstrating six steps of handwashing in correct sequence for two minutes
• Demonstrating steps for normal care after birth (tying umbilical cord, cleaning eyes, and skin-to-skin contact)
**Hypothermia prevention**
• Demonstrating four steps to prevent heat loss in a baby
• Demonstrating measurement of axillary temperature of baby─correct positioning and timing
• Demonstrating correct technique for wrapping the newborn baby
**Kangaroo mother care (KMC)**
• Demonstrating correct steps for positioning of the baby for KMC
**Breastfeeding**
• Demonstrating correct steps for positioning of the baby for breastfeeding
